# What Does Routinely Collected Pooled DIALOG, PROM and PREM Data Tell Us?

**DOI:** 10.1192/bjo.2025.10226

**Published:** 2025-06-20

**Authors:** Akshith Shetty, Stuart Spicer, Rahul Bhattacharya

**Affiliations:** 1 ELFT, London, United Kingdom; 2 University of Plymouth, Plymouth, United Kingdom

## Abstract

**Aims:** The DIALOG scale has been implemented as a routine patient outcome and experience measure (PROM/PREM) in East London Foundation Trust (EL FT). We used large routinely collected DIALOG data to assess impact of treatment across different domains of life and whether the impact of treatment changed with Community Mental Health Transformation CMH (NHS Long Term Plan). We also carried out secondary disaggregation analysis of pooled data based on protected characteristics interrogating through an equity lens.

**Methods:** EL FT had commissioned University of Plymouth for the review of CMH transformation. Anonymised pooled data set was obtained from the electronic patient records that were collected as a part of routine clinical practice. DIALOG (PROM and PREM) scores captured routinely from CMH services in ELFT over two time periods (2018–19 and 2021–22) were collected for this purpose.

The anonymised and pooled data was linked with stages of treatment e.g. assessment, review and at discharge and protected characteristics (age, gender, ethnicity and a proxy of social deprivation).

14,813 DIALOG scores from 6,538 unique patients were identified. We analysed each domain of DIALOG separately and the numbers of return of scores on each domain varied depending on response rate. We compared domain based descriptive statistical analyses of mean pooled DIALOG scores looking at means across a range of variables for each domain and then conducted a series of multiple regressions for each of the DIALOG domains, to control for multiple variables together

**Results:** Our results showed that service user satisfaction in each domain improved with treatment stage (from assessment to review to discharge) reaching statistical significance at each stage. There were minor differences between the two time periods (2018–19 and 2021–22) in a few domains. There was variation in outcomes across ethnicity, age and gender in a few domains.

**Conclusion:** Large data sets of routinely collected DIALOG data offer valuable insight into the needs of the local population and impact of treatment. Assessment of the impact of the CMH service transformation was confounded by the pandemic. Disaggregated data on protected characteristics reveal interesting and useful information about experiences and outcomes of different population groups over time. Our study also validates DIALOG as a quality of life measure and patient experience measure scale that is sensitive to measure change. It affirms the value and depth that intelligence routine outcome data gathering can offer both to measure change as well as offering an assessment of population need.

